# Fatty liver biomarkers and insulin resistance indices in the prediction of non‐alcoholic fatty liver disease in Ghanaian patients

**DOI:** 10.1002/edm2.456

**Published:** 2023-10-09

**Authors:** A. S. Bockarie, Y. A. Nartey, P. Nsiah, E. K. M. Edzie, D. Tuoyire, S. Acquah, S. Eliason, B. Nkum

**Affiliations:** ^1^ Department of Internal Medicine & Therapeutics University of Cape Coast Cape Coast Ghana; ^2^ Department of Medicine Cape Coast Teaching Hospital Cape Coast Ghana; ^3^ Department of Chemical Pathology University of Cape Coast Cape Coast Ghana; ^4^ Department of Radiology University of Cape Coast Cape Coast Ghana; ^5^ Department of Community Medicine University of Cape Coast Cape Coast Ghana; ^6^ Department of Medical Biochemistry University of Cape Coast Cape Coast Ghana; ^7^ Department of Medicine Kwame Nkrumah University of Science and Technology Kumasi Ghana

**Keywords:** fatty liver index, Ghana, hepatic steatosis index, non‐alcoholic fatty liver disease, triglyceride glucose index

## Abstract

**Background:**

Scant West African data on non‐alcoholic fatty liver disease (NAFLD) means there is little representation of this population in the modelling used to derive biomarkers and predictive indices for risk stratification of patients for the presence of hepatic steatosis. This study evaluates the performance of the fatty liver index (FLI), hepatic steatosis index (HSI) and triglyceride‐glucose (TyG) index and its derivatives in predicting ultrasound detected NAFLD in a locally resident population of Ghanaian participants.

**Methods and Findings:**

A post hoc analysis of data from a cross sectional assessment of NAFLD and cardiovascular risk was performed. Data from 210 participants without significant alcohol intake, or secondary causes of fatty liver and not on steatogenic drugs was evaluated. A structured questionnaire had been used to collect demographic data, medical and drug history. Anthropometry, blood sampling for liver chemistry and fasting lipids were performed. Hepatic steatosis was detected by ultrasonography. A retrospective analysis involving multivariate binary logistic regression assessed FLI, HIS, TyG (and its derivatives) as predictors of NAFLD with *p* < .05 considered statistically significant. Sensitivity, specificity, predictive values, likelihood ratios were calculated and accuracy of the proxies evaluated from area under the receiver operating characteristics curve (AUROC).

All the biomarkers and indices were significantly associated with NAFLD (*p* ≤ .001). All the lipid and fatty liver indices assessed performed acceptably as predictors of NAFLD. FLI (AUC = 0.8, 95% CI [0.74–0.87]), TyG‐WC (AUC = 0.81, 95% CI [0.75–0.88]) and TyG‐WHtR (AUC = 0.81, 95% CI [0.74–0.88]) performed best at predicting NAFLD. Whilst in all cases the markers had good specificity (>90%) they lacked sufficient sensitivity with FLI having the highest sensitivity of 36.7%. Their overall accuracy was greater than 70% in each case.

**Conclusion:**

The overall accuracy of HSI, FLI, TyG index and its derivatives (TyG WHtR, TyG BMI, TyG WC) was acceptable for predicting NAFLD in this population. Given their performance in this study and in light of their low cost, accessibility, easy interpretation and non‐invasive nature; they are suitable tools for screening in the Ghanaian population.

## BLURB FOR ETOC

This study evaluates the performance of the fatty liver index, hepatic steatosis index (HSI) and triglyceride‐glucose (TyG) index and its derivatives in predicting ultrasound detected NAFLD in a locally resident population of Ghanaian participants. HSI, FLI biomarkers and TyG, TyG WHtR, TyG BMI, TyG WC indices were significantly associated with NAFLD (*p* = .001). The overall accuracy of the biomarker and indices was found to be acceptable for predicting NAFLD in this Ghanaian Population.

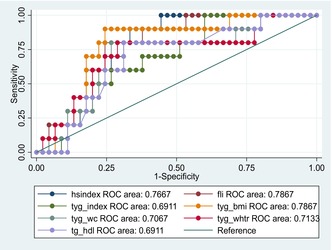

## 1 INTRODUCTION

With the growing recognition of non‐alcoholic fatty liver disease (NAFLD) as a precursor for chronic hepatic diseases and as a significant risk factor for extra‐hepatic, systemic disease outcomes,^1^ there have been growing efforts to develop efficient, cost‐effective, minimally invasive, accurate methods for screening patients for NAFLD. While liver biopsy for histopathology is continually upheld as the gold standard, it is acknowledged that this is not without its shortcomings, including sampling errors, high cost, risk of complications and variability in pathological diagnosis.^2^ Ultrasonography, despite its shortcomings in detecting steatosis under 20%, remains relevant in the diagnosis of NAFLD.^3^, ^4^

Efforts to identify useful risk stratifying proxies using blood biomarkers and anthropometric measurements have led to the development of numerous indices that are proposed to predict the presence or otherwise of hepatic steatosis. The scant West African data on NAFLD means there is little by way of representation of this population group in the modelling used in deriving these predictive indices. While the utility of these indices has been validated to various degrees in populations of European descent^5^, ^6^, ^7^ and Asian populations,^8^, ^9^ to our knowledge, no study has specifically assessed their comparative performance in predicting ultrasound defined NAFLD in a wholly West African resident population. The fatty liver index (FLI)^10^ and hepatic steatosis index^11^ were developed as potential predictors of NAFLD and they have performed better than most other biomarkers in this respect.^12^ The triglyceride‐glucose (TyG) index^13^ is a lipid related index that was initially developed to aid in the assessment of insulin resistance. The role of insulin resistance in the pathogenesis of hepatic steatosis forms part of the rationale behind the increasing use of the terminology metabolic dysfunction‐associated steatosis liver disease (MASLD) in patients with steatosis and features of cardiometabolic dysfunction.^14^ However, the TyG index (together with its derivatives) has joined the FLI and hepatic steatosis index to become some of the most frequent non‐invasive tests or biomarkers utilised in evaluating the likelihood of fatty liver disease.^15^

Developed in the Italian Dionysos nutrition and liver study,^10^ and validated against ultrasonography, the use of FLI as a simple yet accurate marker for NAFLD has been widely assessed and its use endorsed by professional bodies.^4^ Its validity is, however, still debated, and a recent large meta‐analysis found that while it is effective in stratifying the risk of NAFLD, it failed to sufficiently diagnose or exclude NAFLD.^16^ Derived from the findings of a large cross‐sectional study in South Korea, the HSI is easier to calculate and does not require the input of lipid related variables. As such, it potentially represents an economical alternative to FLI.^11^

NAFLD is increasingly recognised as a growing threat in Sub‐Saharan Africa^17^, ^18^ with the age‐standardised prevalence of NAFLD ranging from 5.0%–7.5% to 10.1%–12.5%. Global Burden of Disease data estimated an annual percentage change (EAPC) increase in Western sub‐Saharan Africa of 0.69 (95% CI [0.63–0.75]), with Ghana having an estimated annual percentage increase of 1.26–1.5.^19^ There are already some indications that there are differences in the utility of non‐invasive tests (NITs) for NAFLD between patients of Ghanaian descent living in Europe and other ethnicities living in the same region.^20^ This study aimed at evaluating the performance of insulin resistance indices and steatosis biomarkers derived from various clinical, anthropometric and biochemical measures in predicting NAFLD in a population of Ghanaian participants resident in Ghana.

## 2 MATERIALS AND METHODS

### 2.1 Study Design, Site and Sampling

This work is a sub study of a cross sectional assessment of NAFLD and cardiovascular risk carried out over a 10‐month period from April 2016 to February 2017. Utilising a consecutive sampling approach, 310 patients were consented for the study. Eighty two met one or more of the exclusion criteria. 18 failed to complete all required aspects of the study resulting in a final group of 210 recruited patients from among adult patients (>18 years) of Ghanaian descent who were attending general medical outpatient clinics at the Cape Coast Teaching Hospital, a tertiary health facility in Cape Coast, Central Region, Ghana.

### 2.2 Exclusion criteria

The 210 participants had been screened for significant alcohol intake using the Alcohol Use Disorders Identification Test^21^ (AUDIT) and those with scores of ≥7 or self‐reported alcohol consumption of >14 units per week for women and 21 units for men were excluded from the study. Patients diagnosed with hepatitis B, C or HIV infection or known liver disease were excluded. Patients suffering from thyroid disease, chronic hemolytic disorders, malignancy or on chemotherapy drugs, corticosteroids, valproate or antiretroviral drugs were also excluded.

### 2.3 Ethical Considerations

Ethical approval for the recruitment of patients into the NAFLD and cardiovascular risk study was obtained from the Institutional Review Board of the University of Cape Coast (UCCIRB/CHAS/2015/19). Permission was also obtained from the Cape Coast Teaching Hospital for the study (CCTH/G/002/93‐15). Informed consent was obtained from all participants and documented. The recommendations guiding physicians in biomedical research involving human subjects issued by the World Medical Declaration of Helsinki (2013)^22^ were applied during that study. This paper presents a poc hoc analysis of anonymised data generated by that study.

### 2.4 Data collection

Demographic data, medical and drug histories had been obtained using a structured questionnaire. Significant use of alcohol was ruled out based on both self‐reported usage as well as the administration of the AUDIT questionnaire.^21^ Height was measured to the nearest 0.5 cm using a stadiometer (Seca® mechanical column scale with stadiometer), and weight in light clothes was measured to the nearest 0.1 kg using an electronic scale (Omron® BHF‐510). Body mass index (BMI) was computed as the weight in kilogrammes divided by the square of the height in metre (kg/m^2^). Waist circumference (WC) was measured in centimetres with an inelastic tape measure at the midpoint between the lower margin of the last rib and the top of the iliac crest. Waist‐to‐height ratio (WHtR) was computed by dividing the waist by the height. Hip circumference was measured around the widest portion of the buttocks. Waist‐to‐hip ratio (WtH) was computed by dividing WC by the hip circumference.

### 2.5 Sample collection, analysis and imaging

All study subjects had under gone phlebotomy after an overnight fast (of at least 8 hours). Hepatitis B and C serological status was ascertained by commercially available Micropoint® Hepatitis B surface antigen Gold Rapid and Micropoint® hepatitis C Gold Rapid Test kits, respectively, for the qualitative detection of hepatitis B surface antigen and antibodies to HCV in the serum. Serum was used for the measurement of transaminases and lipids in a Chemistry auto‐analyzer (ChemWell® from Awareness Technology) using Randox laboratories clinical chemistry kits. Total cholesterol analysis was carried out using an enzymatic colorimetric test, the CHOD‐PAP method, high density lipoprotein cholesterol (HDL) by precipitation method and triglyceride (TG) estimation employed enzymatic hydrolysis of TG with lipases. Low density lipoprotein cholesterol (LDL) was calculated using Friedwald's formula: LDL = (Total Cholesterol − HDL)‐TG/2.2 as all the levels of TG were below 5.0 mmol/L.

Abdominal ultrasound scans had been carried out by a radiologist with a Philips ClearVue 350® ultrasound machine to identify sonographic evidence of fatty liver, utilising the criteria set by Hamaguchi et al which were a brighter hepatic echo pattern in comparison to the kidneys, deep attenuation and vascular blurring; present either alone or in combination (p 2710).^23^

### 2.6 Biomarkers and Indices

The selected biomarkers and indices were calculated as follows.

Hepatic steatosis index (HSI) = 8 × (ALT/AST ratio) + BMI* (+2, if female; +2, if diabetes mellitus is present)

$$\begin{array}{cc} \text{Fatty liver index}\;\left(\mathrm{FLI}\right)= & \left[e^{\left(0.953\times \ln\left(\text{triglycerides}\right)+0.139\times \mathrm{BMI}+0.718\times \ln\left(\text{γGTP}\right)+0.053\times \mathrm{WC}-15.745\right)}\right]/\\ & \left[1+e^{\left(0.953\times \ln\left(\text{triglycerides}\right)+0.139\times \mathrm{BMI}+0.718\times \ln\left(\text{γGTP}\right)+0.053\times \mathrm{WC}-15.745\right)}\right]\times 100 \end{array}$$

$$\text{Triglyceride}-\text{Glucose}\;\left(\mathrm{TyG}\right)\;\text{index}=\mathrm{Ln}\;\left[\text{Fasting triglyceride}\;\left(\mathrm{mg}/\mathrm{dL}\right)\times \text{fasting glucose}\;\left(\mathrm{mg}/\mathrm{dL}\right)\right]/2$$

TyG−BMI=TyG×BMI

TyG−WC=TyG×WC

TyG−WHtR=TyG×WHtR

$$\text{Triglyceride}/\mathrm{HDL}\;\text{ratio}\;\left(\mathrm{TG}-\mathrm{HDL}\right)=\mathrm{TG}\;\left(\text{mmol}/l\right)/\mathrm{HDL}\;\left(\text{mmol}/L\right)$$

### 2.7 Data analysis

Data were entered in Microsoft excel® and exported to Stata®, version 15; StataCorp, software for analysis. Descriptive statistics was used to describe measures of central tendencies, frequencies and percentages. Pearson's χ^2^‐test was used to find associations between categorical variables. Student *t*‐test (independent two‐sample) was used to compare the means of scale variables with two levels of category. Cohen's d, a standardised effect size measure, was used to determine the magnitude of the difference between the means of the independent variables, with a Cohen's *d* ≥ 0.8 indicative of a large effect size.

Binary logistic regression was used to determine the relationship between relevant clinical, anthropometric parameters, insulin resistance indices and steatosis biomarkers and the presence of NAFLD. Those variables with a *p* ≤ .25 were considered for multivariate binary logistic regression analysis. Models were adjusted for relevant sociodemographic and other risk factor variables. A *p* < .05 was considered statistically significant. The accuracy, sensitivity, specificity, positive and negative predictive values as well as positive and negative likelihood ratios and AUROC were calculated for each insulin resistance index and steatosis biomarker. AUROC of ≥0.7 to <0.8 was considered acceptable, ≥0.8 to <0.9 was considered good, and ≥0.9 was considered excellent. Post hoc analyses on the fitness of the logistic regression models were conducted using the Hosmer‐Lemeshow goodness‐of‐fit test.

## 3 RESULTS

### 3.1 The study population

The 210 participants were predominantly female, with a female‐to‐male ratio of 2.6:1 with a median age of 54 years (IQR 42–61). The mean BMI, WC and waist‐to‐hip ratio of the study participants were 28.29 kg/m^2^ (SD = 8.28), 94.34 cm (SD = 17.92) and 0.90 (SD = 0.12) respectively (Table 1). Females had a higher mean BMI than males (29.32 kg/m^2^ vs. 25.69 kg/m2, *p* = .004) as well as higher WC (96.28 cm versus 89.27 cm, *p* = .01). The prevalence of obesity, hypertension, diabetes, dyslipidemia and metabolic syndrome among the participants was 76 (37.44%), 125 (59.52%), 35 (16.67%), 31 (14.76%) and 106 (50.47%), respectively.

**TABLE 1:** Characteristics of study participants.

Parameter	Entire population Mean ± SD
BMI (kg/m^2^)	28.29 ± 8.28
Systolic BP (mmHg)	130.82 ± 20.77
Diastolic BP (mmHg)	82.97 ± 14.86
FPG (mmol/l)	6.34 ± 2.97
ALT (U/L)	13.50 ± 7.95
AST (U/L)	23.68 ± 8.84
GGT (U/L)	28.73 ± 24.04
Cholesterol (mmol/L)	5.13 ± 1.44
TG (mmol/L)	1.08 ± 0.65
LDL (mmol/L)	3.90 ± 4.05
HDL (mmol/L)	1.15 ± 0.29
WtH	0.90 ± 0.12
WtHR	0.59 ± 7.95

Abbreviations: ALT, alanine aminotransferase; AST, aspartate aminotransferase; BMI, body mass index; BP, blood pressure; Chol, total cholesterol; FPG, fasting plasma glucose; GGT, gamma‐glutamyl transferase; HDL, high‐density lipoprotein cholesterol; LDL, low‐density lipoprotein cholesterol; SD, standard deviation; TG, triglyceride.

A total of 52 patients (24.8%) met the ultrasonographic criteria for diagnosis of fatty liver, 10 of whom were males (17.2%) and 42 females (27.6%). There were significant differences between those with NAFLD and those without sonographic evidence of NAFLD in relation to age, waist‐to‐hip ratio, waist‐to‐height ratio, diastolic and systolic blood pressures, BMI, fasting plasma glucose (FPG), TG and HDL‐C with the NAFLD group generally having higher mean values than the non‐NAFLD group (all *p* < .05; Table 2). The resulting Cohen's d value for the differences in mean BMI (*d* = .88) and WHtR (*d* = .98) between the groups suggested a large size effect. These differences in means were, however, not significant for laboratory characteristics like ALT, total cholesterol and HDL‐C. Participants with NAFLD had a slightly lower mean LDL‐C but this was also not statistically significant (3.35 ± 1.51, *p* = .59) as shown in Table 2.

**TABLE 2:** Demographic, anthropometric, clinical and laboratory characteristics of study subjects with and without sonographic evidence of NAFLD.

	NON‐NAFLD (*n* = 158)	NAFLD (*n* = 52)		
Parameter	Mean ± SD	Mean ± SD	*p*‐value	Cohen's d
BMI (kg/m^2^)	26.01 ± 7.34	31.95 ± 9.34	<.001	.88
Age (years)	50.13 ± 15.66	54.83 ± 10.30	.04	.32
Waist‐to‐hip ratio	0.89 ± 0.14	0.93 ± 0.06	.03	.36
Waist‐to‐height ratio	0.57 ± 0.10	0.67 ± 0.09	<.001	.98
Systolic (mmHg)	126.45 ± 20.31	137.94 ± 20.01	.001	.57
Diastolic (mmHg)	80.00 ± 15.34	87.27 ± 12.02	.001	.52
FPG (mmol/L)	5.72 ± 2.53	6.93 ± 3.80	<.001	.70
ALT (U/L)	11.78 ± 7.44	13.61 ± 9.21	.070	.30
AST (U/L)	22.24 ± 8.03	24.63 ± 10.84	.052	.32
GGT (U/L)	20.57 ± 18.42	32.75 ± 33.86	<.001	.71
Chol (mmol/L)	5.66 ± 4.59	5.45 ± 1.4	.73	.05
TG (mmol/L)	1.13 ± 0.65	1.22 ± 0.54	<.001	.66
LDL (mmol/L)	3.49 ± 4.59	3.35 ± 1.51	.590	.07
HDL (mmol/L)	1.10 ± 0.29	1.14 ± 0.29	.470	.12

Abbreviations: ALT, alanine aminotransferase; AST, aspartate aminotransferase; BMI, body mass index; BP, Blood pressure; Chol, total cholesterol; FPG, fasting plasma glucose; GGT, gamma‐glutamyl transferase; HDL, high‐density lipoprotein cholesterol; Low‐density lipoprotein; NAFLD, Non‐alcoholic fatty liver disease; SD, standard deviation; TG, triglyceride.

### 3.2 Clinical and anthropometric parameters as predictors of NAFLD

Increasing BMI (COR 2.77 95% CI [1.80–4.23]), WC (COR 11.01 95% CI [2.57–47.12]), WHtR (COR 14.75 95% CI [2.0–110.96]) and WtH ratio (COR 4.39 95% CI [1.64–11.70]) were predictors of NAFLD on bivariate analysis. In the case of WtH ratio, WHtR and WC these failed to meet the benchmark for significance after adjustment and controlling for potential confounders in multivariate models (Table 3). BMI remained a predictor of NAFLD after adjustment and controlling for covariates (AOR = 2.14, 95% CI [1.18–3.88]). The odds of NAFLD among participants with hypertension (COR = 1.96, 95% CI [0.99–3.86]), diabetes (COR = 0.88 95% CI [0.37–2.08]) and dyslipidemia (COR = 0.87, 95% CI [0.35–2.15]) were not significant compared to those without (Table 3).

**TABLE 3:** Bivariate and multivariate binary logistic regression of some clinical and anthropometric parameters as predictors of NAFLD.

Parameter	COR (95% CI)	*p‐*Value	AOR (95% CI)	*p‐*Value	Model 1 AOR (95% CI)	*p‐*Value	Model 2 AOR (95% CI)	*p‐*Value
BMI	2.77 (1.80–4.23)	<.001	2.16 (1.33–3.50)	.002	2.12 (1.28–3.49)	.003	2.14 (1.18–3.88)	.01
WtH	4.39 (1.64–11.70)	.003	2.30 (0.78–6.78)	.13	2.05 (0.65–6.46)	.22	1.60 (0.35–7.20)	.54
WC	11.01 (2.57–47.12)	.001	2.67 (0.46–15.30)	.27	2.93 (0.49–17.48)	.24	4.68 (0.42–51.74)	.21
WHtR	14.75 (2.0–110.96)	.001	1.84 (0.89–18.09)	.60	1.56 (0.15–6.08)	.71	1.67 (0.16–17.80)	.67
Hypertension	1.96 (0.99–3.86)	.05	‐	‐	‐	‐	‐	‐
Diabetes	0.88 (0.37–2.08)	.78	‐	‐	‐	‐	‐	‐
Dyslipidemia	0.87 (0.35–2.15)	.76	‐	‐	‐	‐	‐	‐

*Note*: **MODEL 1:** sociodemographic variables **(age, sex, education, employment and marital status)** were adjusted for this model. **MODEL 2:** risk factor variables **(alcohol consumption, smoking, triglycerides, high density lipoprotein and medication)** of fatty liver was adjusted for this model. *p* < .05 was considered significant.

Abbreviations: AOR, adjusted odds ratio; BMI, body mass index, COR, crude odds ratio; NAFLD, non‐alcoholic fatty liver disease; WC, waist circumference; WHtR, Waist‐to‐height ratio; WtH, waist‐to‐hip ratio.

### 3.3 Liver steatosis indices and insulin resistance related biomarkers as predictors of NAFLD

Participants with NAFLD had significantly higher mean lipid and related metabolic indices than participants without NAFLD on ultrasonography (all *p* ≤ .001). With the exception of the triglyceride‐to‐high‐density lipoprotein cholesterol ratio, all the variables in this category demonstrated a large effect size (all Cohen's *d* > .8). This is shown in Table 4.

**TABLE 4:** Comparison of means of lipid and related metabolic indices as predictors of NAFLD for subjects with and without sonographic evidence of NAFLD.

	Non‐NAFLD (*n* = 158)	NAFLD (*n* = 52)		
Biomarker/Indices	Mean ± SD	Mean ± SD	*p*‐value	Cohens d
HSI	32.37 ± 6.82	38.50 ± 6.54	<.001	.85
FLI	24.24 ± 25.23	54.99 ± 28.86	<.001	1.2
TyG index	4.06 ± 0.28	4.34 ± 0.30	<.001	.94
TyG BMI	110.16 ± 34.71	142.61 ± 44.47	<.001	.87
TyG WC	373.92 ± 70.63	458.45 ± 71.40	<.001	1.2
TyG WHtR	2.32 ± 0.47	2.85 ± 0.45	<.001	1.1
TG/HDL	0.92 ± 0.55	1.22 ± 0.64	.001	.53

Abbreviations: FLI, Fatty liver index; HSI, Hepatic steatosis index; NAFLD, Non‐alcoholic fatty liver disease; TG/HDL, triglyceride to high‐density lipoprotein cholesterol ratio; TyG index, triglyceride‐glucose index; TyG‐BMI, triglyceride glucose‐body mass index; TyG‐WC, triglyceride glucose‐waist circumference; TyG‐WHtR, triglyceride glucose‐ waist‐to‐height ratio.

After controlling for sociodemographic and clinical variables (Model 3) the logistic regression analysis demonstrated that FLI (AOR = 1.04, 95% CI [1.01–1.09], *p* = .03), and TG/HDL ratio (OR = 0.28, 95% CI [0.09–0.86], *p* = .03) were predictors of NAFLD in this study population, as was TyG index (OR = 36.25, 95% CI [2.26–581.57], *p* = .01) although with a much greater degree of uncertainty (Table 5).

**TABLE 5:** Bivariate and multivariate binary logistic regression of lipid related and fatty liver indices as predictors of NAFLD.

					Model 1		Model 2		Model 3	
Indices/Biomarker	COR (95% CI)	*p*‐value	AOR (95% CI)	*p*‐value	AOR (95% CI)	*p*‐value	AOR (95% CI)	*p*‐value	AOR (95% CI)	*p*‐value
HSI	1.13 (0.07–1.19)	<.001	0.96 (0.85–1.07)	.45	0.95 (0.84–1.07)	.38	0.95 (0.84–1.08)	.42	0.94 (0.82–1.07)	.35
FLI	1.04(1.02–1.05)	<.001	1.03 (0.10–1.06	.08	1.03 (1.00–1.07)	.05	1.04 (1.00–1.08)	.04	1.04 (1.01–1.09)	.03
TyG index	32.37 (7.93–132.20	<.001	27.72 (2.15–357.27)	.011	34.56 (2.53–472.93)	.01	32.96 (2.17–499.71)	.01	36.25 (2.26–581.57)	.01
TyG BMI	1.03 (1.01–1.04)	<.001	1.00 (0.99–1.02)	.73	1.01 (0.18–1.04)	.62	1.00 (0.99–1.02)	.74	1.00 (0.98–1.03)	.69
TyG WC	1.02 (1.01–1.03)	<.001	1.01 (0.99–1.03)	.51	1.01 (0.98–1.04)	.35	0.99 (0.97–1.02)	.97	1.00 (0.98–1.03)	.77
TyG WHtR	12.79 (5.14–31.84)	<.001	0.79 (0.03–18.16)	.89	0.25 (0.01–11.92)	.48	1.48 (0.04–58.62)	.84	0.59 (0.01–38.78)	.81
TG/HDL	2.22 (1.31–3.76)	<.001	0.37 (0.14–0.98)	.04	0.35 (0.13–0.94)	.04	0.29 (0.09–0.88)	.03	0.28 (0.09–0.86)	.03

*Note*: MODEL 1: sociodemographic variables (age, sex) were adjusted for this model. MODEL 2: smoking, alcohol consumption, systolic and diastolic blood pressure were adjusted for this model. *p* < .05. MODEL 3: age, sex, smoking, alcohol consumption, systolic and diastolic blood pressures were adjusted for this model.

Abbreviations: FLI, Fatty liver index; HSI, Hepatic steatosis index; NAFLD, Non‐alcoholic fatty liver disease; TG/HDL, triglyceride to high‐density lipoprotein cholesterol ratio; TyG index, triglyceride‐glucose index; TyG‐BMI, triglyceride glucose‐body mass index; TyG‐WC, triglyceride glucose‐waist circumference; TyG‐WHtR, triglyceride glucose‐ waist‐to‐height ratio.

The accuracy, sensitivity, specificity, predictive values and likelihood ratios for the lipid and steatosis biomarkers are shown in Table 6. With the exception of TG/HDL ratio, all the lipid and fatty liver indices performed acceptably as predictors of NAFLD with AUC values >0.7. FLI (AUC = 0.8, CI [0.74–0.87]), TyG‐WC (AUC = 0.81, CI [0.75–0.88]) and TyG‐WHtR (AUC = 0.81, CI [0.74–0.88]) performed best at predicting NAFLD. While in all cases the markers had good specificity (>90%) they lacked sufficient sensitivity, with FLI performing best at a sensitivity of 36.7% (Table 6). With the exception of the TyG BMI, the *p* values for all the models using the Hosmer Lemeshow test were >.05 indicating a good fit for the models.

**TABLE 6:** Performance of logistic models and goodness of fit.

BIOMARKER/INDICES	Sensitivity %	Specificity %	PPV %	NPV %	Accuracy %	PLR	NLR	Hosmer Lemeshow gof	AUROC (95% CI)
COR Model‐HSI	10.2	95.45	41.67	76.96	74.88	2.24	0.94	0.68	0.74 (0.66–0.81)
COR Model‐FLI	36.73	91.61	58.06	82.08	74.43	4.38	0.69	0.65	0.80 (0.74–0.87)
COR Model TyG index	18	94.84	52.94	78.19	76.1	3.49	0.86	0.6	0.76 (0.68–0.82)
COR Model TyG BMI	14	97.42	63.64	77.84	77.07	5.43	0.88	0.41	0.78 (0.71–0.85)
COR Model TyGWC	30.61	92.9	57.69	80.9	77.94	4.31	0.75	0.69	0.81 (0.75–0.88)
COR Model TyG WHtR	26.53	94.19	59.09	80.22	77.9	4.57	0.78	0.94	0.81(0.74–0.88)
COR Model TG/HDL	6	98.05	50	76.26	77.9	3.08	0.96	0.36	0.66 (0.56–0.74)

Abbreviations: AOR, Adjusted odds ratio; AUC, Area under ROC curve; COR, Crude odds ratio; FLI, Fatty liver index; gof, goodness of fit; HSI, Hepatic steatosis index; NLR, Negative likelihood ratio; NPV, Negative predictive value; PLR, Positive likelihood ratio; PPV, Positive predictive value; TG/HDL‐C, triglyceride to high‐density lipoprotein cholesterol ratio; TyG index, triglyceride‐glucose index; TyG‐BMI, triglyceride glucose‐body mass index; TyG‐WC, triglyceride glucose‐waist circumference; TyG‐WHtR, triglyceride glucose‐waist‐to‐height ratio.

**FIGURE 1:**
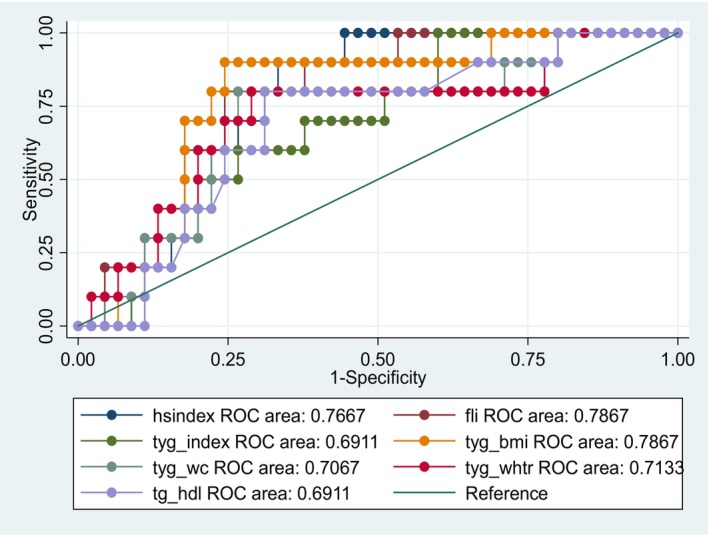
ROC curve for males.

**FIGURE 2:**
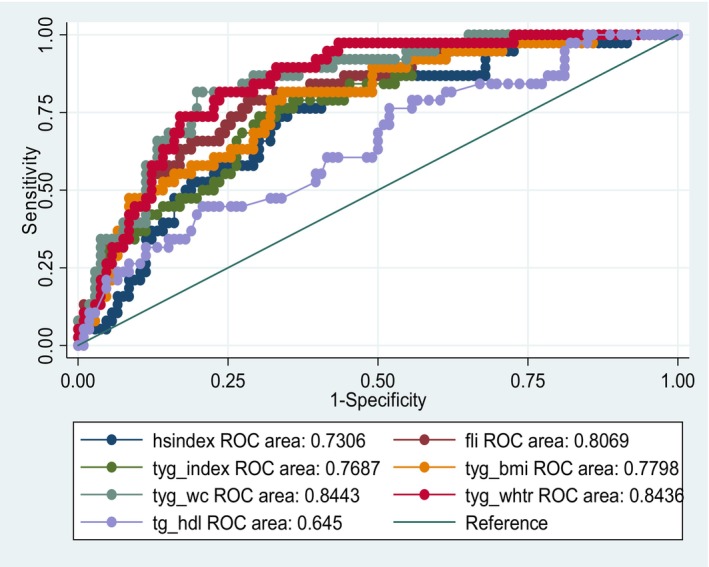
ROC curve for females.

When the data were segregated by sex the overall trend among the predictors was maintained in females, who made up the majority of participants. FLI performed slightly better in females than it had in males. In the solely male group, TyG‐BMI and FLI were the best predictors of NAFLD with an AUC = 0.79 (Figures 1 and 2).

## 4 DISCUSSION

This analysis assessed the performance of various biomarkers derived from clinical, anthropometric and lipid related measures as predictors of NAFLD among an undifferentiated pool of participants from medical outpatient clinics at a teaching hospital in Ghana. Our results demonstrated that variables such as waist‐to‐hip ratio, waist‐to‐height ratio, diastolic and systolic blood pressures, BMI, fasting plasma glucose (FPG) and TG were significantly associated with NAFLD. Postulated to be driven by insulin resistance among other things,^24^ the relationship between fatty liver and obesity has been continually reaffirmed.^25^, ^26^ Despite the relationship between FPG and NAFLD, diabetes mellitus was not found to be a predictor of steatosis in our patient cohort, which is at variance with that of a Turkish study.^27^ This might have to do with the common use of medication such a metformin and pioglitazone among diabetics which are known to affect fatty liver. Our work found a significant association between NAFLD and the anthropometric measurements we assessed, which included BMI, WtH and WHtR. As a predictor of NAFLD, BMI remained significant even after adjustment and after controlling for smoking, triglycerides, high density lipoprotein and medication. We found no studies that had previously evaluated BMI as a predictor of NAFLD in West Africa. However, the association between BMI and NAFLD has been suggested by findings in other sub regional studies. In Nigeria^28^, ^29^ and in Sudan,^30^ BMI, was found to be significantly higher among subjects with NAFLD than in those without NAFLD. Among diabetics^31^ and participants with HIV^32^ in Nigeria with NAFLD; however, this difference in BMI was not found to be significant probably because of the effect of their underlying diseases and the associated treatments in those sub populations. One previous Nigerian study assessing the utility of novel indices like TyG index, TyG WC, TyG BMI and TyG WHtR evaluated their efficacy as predictors of metabolic syndrome (MetS) and it was found that all these indices significantly identified metabolic syndrome in the participants.^33^ Despite the lack of previous studies assessing the performance of these novel insulin resistance indices in predicting NAFLD in a West African population, the frequently demonstrated association between NAFLD and MetS^34^ makes the relationship found between those indices and NAFLD in our study population plausible.

The current study also demonstrated that the composite biomarkers (HSI, FLI) and lipid related indices (TyG index, TyG WC, TyG WHtR, TG/HDL) reviewed were associated with NAFLD in our study participants. Using the same cut‐offs suggested by Bedogni et al in the initial study,^10^ FLI had slightly better specificity in our study (91.61%) than in the initial study (86%) and as a predictive modality, it was found to be good in this study (AUC 0.8, CI [0.74–0.87]). A larger European study that also utilised ultrasonography found that FLI score was associated with NAFLD (AOR 1.05, 95% CI 1.04–1.05, *p* < .001),^5^ which is similar to what was found on multivariate analysis in this study (AOR 1.04, 95% CI 1.01–1.09 *p* = .03) (Table 5, Model 3. For the prediction of NAFLD, FLI performed just as well among our study participants (AUC 0.80 95% CI [0.74–0.87]) as it had among those European participants (AUC 0.813 95% CI [0.797–0.830]).^5^ Despite the observation of adequate specificities of 85% and 91.6% respectively, for the European study^5^ and the current one, sensitivity in the European population was much higher than was found in our study (61% vs. 36.7%, respectively). While the low sensitivity of FLI for detecting NAFLD in our population (36.7%) might raise questions about its suitability as a screening tool for patients at risk of NAFLD in our local context, the proportion of overweight and obese participants should be taken into account. FLI has been found to be a poor predictor of steatosis in obese patients.^35^ Approximately one third of our participants were obese (37.44%) with a mean population BMI of 28.29 kg/m^2^ (SD ± 8.28). This may partly explain the low sensitivity of FLI in identifying those with NAFLD in this study. The NAFLD in Healthy Life in an Urban Setting (HELIUS) study suggested ethnic differences in the utility of biomarkers in detecting NAFLD, comparing participants of Ghanaian descent with those of Dutch, Moroccan and South Asian descent all living in Europe.^19^ That study found Ghanaians also had the lowest steatosis scores based in FLI and the lowest adjusted odds ratio for elevated Controlled Attenuation Parameter (a measurement of steatosis on transient elastography) score (0.18, 95% CI 0.09–0.37) compared to other ethnicities, making FLI relatively less predictive in that migrant Ghanaian population.^19^ No explanation was given for these differences, but it raised a question that this study attempts to answer relating to the performance of these non‐invasive tests in the Ghanaian population.

Several works have reviewed the performance of predictive fatty liver biomarkers for the presence of steatosis. Despite the variations in performance of these biomarkers within different studies, there are widely noted trends. A French study found a relatively poor predictive utility of some biomarkers for NAFLD as diagnosed by ultrasonography, their AUCs were 0.56 (0.48–0.64) for FLI, 0.65 (0.58–0.73) for HSI, and 0.63 (0.54–0.71) for TyG.^7^ Although we cannot directly compare the performance of these variables between studies, we describe our finding of 0.8 (0.74–0.87) for FLI as good (AUC 0.8–0.9), and 0.74 (0.66–0.81) for HSI, 0.76 (0.68–0.82) for TyG index as acceptable (AUC >0.7) in our study. In a separate study among Japanese subjects, the AUCs of all anthropometric and lipid‐related indices were greater than 0.5, indicating that all have certain predictive values for NAFLD.^8^ This finding mirrors our own, with TG HDL in females (AUC = 0.645) being the least predictive in our study. In that Japanese study, TyG index‐related parameters had overall good predictive values [0.79–0.81] for TyG index, 0.84 [0.87–0.89] for TyG BMI, 0.88 [0.88–0.89] for TyG WC and 0.87 [0.87–0.88] for TyG WHtR) which is similar to our finding that TyG related indices performed well in our population. The value of TyG index as a novel biomarker is highlighted in the findings of two meta‐analyses. Ling et al demonstrated a strong positive association and a significant dose–response relationship between TyG index and NAFLD in a meta‐analytic study involving 105,365 participants from 12 independent studies.^36^ In that meta‐analysis, TyG index performed better in females than in males, a finding similar to that of our study. In the second meta‐analysis, which involved 121,975 participants from 17 studies, higher TyG index was found to be an independent predictor of NAFLD.^15^ No African data were included in either of the meta‐analyses cited. Overall, in our study TyG‐WC and TyG‐WHtR performed best at predicting NAFLD, adding credence to the argument that TyG‐related parameters (combining both TyG and anthropometric measures) could be a better predictor of hepatic steatosis compared them with TyG index on its own.^9^, ^37^ In summary, our work has found HSI, FLI and TyG and related indices (TyG index, TyG WC, TyG WHtR satisfactorily predict NAFLD meeting the cut‐off for acceptability (AUROC ≥0.7).

The key limitation of this study is the use of ultrasonography for detecting NAFLD. Although it is less sensitive in detecting steatosis less than 20%, it remains the most widely utilised clinical tool in routinely assessing liver structure^3^ and it continues to appear in many studies assessing non‐invasive markers. The characterisation of hepatic steatosis in this study was limited to its sonographic presence without further characterisation based on the extent of fibrosis or inflammation (steatohepatitis). Being a single facility, hospital‐based study, it is difficult to generalise our findings to a wider community‐based population. It is however our assertion that our study participants represent an important sub population of high‐risk patients in whom these indices could play a critical role for screening and follow‐up. Our predominantly female study participants, though a reflection of the general health seeking behaviour at out health facility, under powers the observations made in males when the data is segregated by sex.

To our knowledge, this study represents the first effort to assess the performance of multiple liver steatosis indices as predictors of NAFLD in a locally resident West African population. It is the first study to assess the relationship between NAFLD and combined lipid related biomarkers in the West African context. This represents the first West African effort to simultaneously assess multiple clinical and anthropometric parameters as predictors of sonographically detected NAFLD. Notwithstanding the acknowledged limitations, this study helps fill a gap in the knowledge surrounding NAFLD in Sub‐Saharan Africa and contributes to the continuing assessment of the utility of biomarkers in identifying NAFLD.

## 5 CONCLUSION

In resource limited settings such as those that prevail in Ghana and more widely across West Africa, widely available and cost‐effective biomarkers, have the potential to have a huge impact and revolutionise screening as well as follow‐up assessment for patients with significant risk factors for NAFLD. New health technologies and strategies for chronic non communicable diseases like NAFLD very often see limited evaluation of their suitability in sub Saharan populations before becoming mainstream. There is a dearth of information regarding the performance of many indices of liver steatosis in Africa, even though there is evidence of the increasing prevalence and growing impact of NAFLD. HSI, FLI, TyG index and its derivatives (TyG WHtR, TyG BMI, TyG WC) were deemed to have good overall accuracy for NAFLD. Given their low cost, wide availability, easy interpretation and non‐invasive nature, we propose them as suitable tools for screening in similar populations of hospital attendants in Ghana. More work is also needed to derive biomarkers with even greater sensitivity for NAFLD in this population.

## AUTHOR CONTRIBUTIONS

**A. S. Bockarie:** Conceptualization (lead); data curation (lead); formal analysis (lead); investigation (lead); methodology (lead); project administration (lead); resources (lead); supervision (lead); writing – original draft (lead); writing – review and editing (equal). **Y. A. Nartey:** Investigation (equal); project administration (equal); supervision (equal); writing – review and editing (equal). **P. Nsiah:** Data curation (equal); formal analysis (equal); investigation (equal); methodology (equal); project administration (equal); supervision (equal); writing – review and editing (equal). **E. K. M. Edzie:** Investigation (equal); methodology (equal); resources (equal); supervision (equal); writing – review and editing (equal). **D. Tuoyire:** Formal analysis (equal); methodology (equal); software (equal); writing – review and editing (equal). **S. Acquah:** Formal analysis (equal); writing – review and editing (equal). **S. Eliason:** Validation (equal); writing – review and editing (equal). **B. Nkum:** Conceptualization (equal); methodology (equal); supervision (equal); validation (equal); writing – review and editing (equal).

## CONFLICT OF INTEREST STATEMENT

All authors declare no conflict of interest.

## Data Availability

The data that support the findings of this study are available from the corresponding author upon reasonable request.
